# Inheritance of rare functional *GCKR* variants and their contribution to triglyceride levels in families

**DOI:** 10.1093/hmg/ddu269

**Published:** 2014-05-30

**Authors:** Matthew G. Rees, Anne Raimondo, Jian Wang, Matthew R. Ban, Mindy I. Davis, Amy Barrett, Jessica Ranft, David Jagdhuhn, Rica Waterstradt, Simone Baltrusch, Anton Simeonov, Francis S. Collins, Robert A. Hegele, Anna L. Gloyn

**Affiliations:** 1 Oxford Centre for Diabetes, Endocrinology & Metabolism, University of Oxford, Oxford OX3 7LE, UK,; 2 National Human Genome Research Institute, National Institutes of Health, Bethesda, MD 20892, USA,; 3 National Center for Advancing Translational Sciences, National Institutes of Health, Rockville, MD 20850, USA,; 4 Departments of Medicine and Biochemistry, Schulich School of Medicine and Dentistry, Robarts Research Institute, University of Western Ontario, London, ON N6A 3K6, Canada,; 5 Institute for Medical Biochemistry & Molecular Biology, University of Rostock, Rostock 18057, Germany and; 6 NIHR Oxford Biomedical Research Centre, ORH Trust, OCDEM, Churchill Hospital, Oxford OX3 7LE, UK

## Abstract

Significant resources have been invested in sequencing studies to investigate the role of rare variants in complex disease etiology. However, the diagnostic interpretation of individual rare variants remains a major challenge, and may require accurate variant functional classification and the collection of large numbers of variant carriers. Utilizing sequence data from 458 individuals with hypertriglyceridemia and 333 controls with normal plasma triglyceride levels, we investigated these issues using *GCKR*, encoding glucokinase regulatory protein. Eighteen rare non-synonymous *GCKR* variants identified in these 791 individuals were comprehensively characterized by a range of biochemical and cell biological assays, including a novel high-throughput-screening-based approach capable of measuring all variant proteins simultaneously. Functionally deleterious variants were collectively associated with hypertriglyceridemia, but a range of *in silico* prediction algorithms showed little consistency between algorithms and poor agreement with functional data. We extended our study by obtaining sequence data on family members; however, functional variants did not co-segregate with triglyceride levels. Therefore, despite evidence for their collective functional and clinical relevance, our results emphasize the low predictive value of rare *GCKR* variants in individuals and the complex heritability of lipid traits.

## INTRODUCTION

The past several years have seen a substantial investment in sequencing projects aimed at exploring the association of rare variants with genetically complex traits such as plasma lipid levels (1). Emerging work suggests that rare non-synonymous variants are important contributors to complex disease susceptibility (2,3). However, studies focusing on rare variants are by their nature limited by sample size (4). Increasing the number of variant carriers available for analysis by grouping rare variants or through family-based studies may increase the power to detect associations. For both approaches, determining the functional consequences of individual rare variants is critical. As the pace of variant discovery currently far outstrips functional characterization, analyses will likely continue to focus on non-synonymous variants classified by computational mutation prediction algorithms. However, the ability of current *in silico* programs to assign pathogenicity correctly to novel non-synonymous variants remains in question (5).

*GCKR*, encoding glucokinase regulatory protein (GKRP), represents a powerful opportunity to investigate the effects of vertically transmitted heterozygous rare variants in relation to plasma triglyceride levels, a complex trait with an estimated heritability of 35–40% (6). A common non-synonymous variant (p.P446L, rs1260326; minor allele frequency = 0.40 in Western Europeans) shows robust evidence for association with triglyceride levels both as a quantitative trait and with hypertriglyceridemia, defined as fasting plasma triglyceride levels above the 95th percentile, utilizing a case–control design (7,8). Additionally, rare non-synonymous *GCKR* variants collectively have been shown to affect triglyceride levels, and also appear enriched in hypertriglyceridemia (3,8). Perhaps most importantly, there is significant biological knowledge about the role of GKRP in the regulation of hepatic glucokinase (GCK), which has allowed the development of assays capable of discerning the effects of non-synonymous variants on protein function. These assays have provided compelling functional evidence biologically consistent with the direction of genetic effects (3,9,10).

The finding that rare variants in genes including *GCKR* are strongly enriched in the cases of hypertriglyceridemia suggests that these variants are collectively relevant to the hypertriglyceridemia phenotype (8). However, the contributions of individual genes and variants remain unclear. We therefore aimed to characterize extensively all rare, non-synonymous *GCKR* variants identified through sequencing of individuals with hypertriglyceridemia and controls with normal plasma triglyceride levels in a range of functional assays, to evaluate the performance of *in silico* tools for assigning pathogenicity, and to investigate the penetrance and heritability of rare *GCKR* variants by exploring co-segregation between heterozygous carriers of functional variants and metabolic phenotypes in families.

## RESULTS

Utilizing a previously described cohort of 458 individuals with hypertriglyceridemia (defined as fasting plasma triglyceride levels above the 95th percentile) and 333 control individuals with normal plasma triglyceride levels, 30 individuals (24 cases, 6 controls) harboring a total of 18 rare (minor allele frequency <0.01) *GCKR* variants were identified by targeted exon sequencing (8). Seven variants (p.R51Q, p.E77G, p.Q234P, p.A519T, p.S183CfsX34, p.T379NfsX36, p.R540X) overlapped with variants reported in a previous population-based sequencing study of *GCKR* (3). These seven variants were observed in both hypertriglyceridemia cases (*n* = 13 individuals) and controls (*n* = 6); the non-overlapping variants (*n* = 11) were therefore each unique to a single individual with hypertriglyceridemia. To provide functional information on uncharacterized *GCKR* variants and to elucidate further the consequences of previously described variants, we carried out functional characterization of all 18 variants.

**Figure 1.**
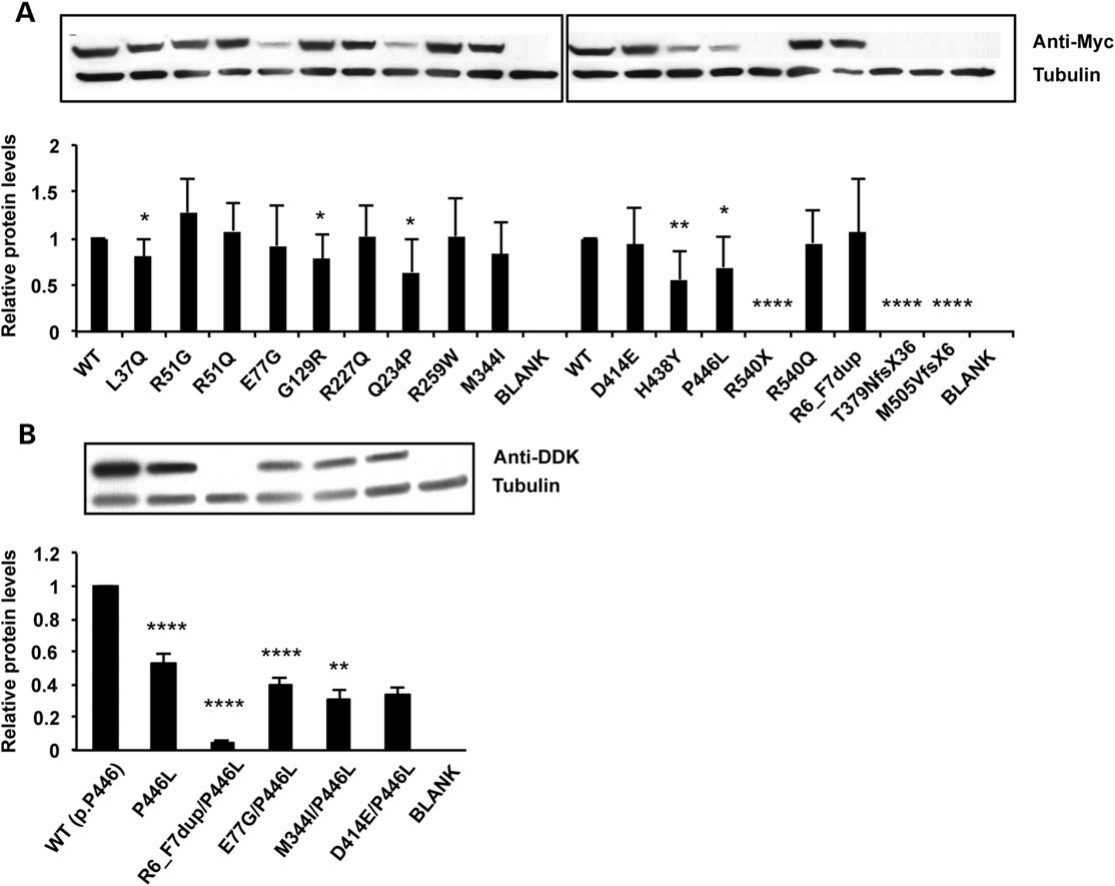
Comparison and quantification of GKRP variant protein expression via western blot analysis. A representative experiment showing protein levels relative to endogenous tubulin for all variants in the p.P446 background (**A**) and p.L446 background (**B**). Quantification of multiple independent transfections [*n* = 4 in (A), *n* = 2 in (B)], performed in duplicate, is displayed underneath. Results shown are mean ± SEM. *P*-values relative to WT *<0.05; **<0.01; ****<0.0001 (unpaired *t-*test).

Three frameshift variants, as well as one nonsense variant, did not show any detectable expression in preliminary cell-based assays (Fig. 1A). One additional variant, a 23-base duplication causing a frameshift near the N-terminus of the protein (c.170_192dup; p.G65MfsX24) was assumed to cause complete loss of function and was not characterized. All 11 *GCKR* missense variants, as well as one in-frame duplication (c.17_22dup; p.R6_F7dup—previously reported as p.Q8_H9insRF), were selected for comprehensive functional characterization. Five variants (p.L37Q, p.G129R, p.Q234P, p.H438Y, p.P446L) resulted in significantly reduced protein expression (Fig. 1A). These results complement previous findings suggesting that the cellular behaviors of p.R51Q-GKRP and p.E77G-GKRP are qualitatively similar to WT-GKRP, while p.Q234P causes a number of defects (3). The phase of the common p.P446L variant could unambiguously be determined in all cases; four variants (p.R6_F7dup, p.E77G, p.M344I and p.D414E) that arose on the minor leucine-446 haplotype were therefore also characterized on the L446 background (Fig. 1B).

**Figure 2.**
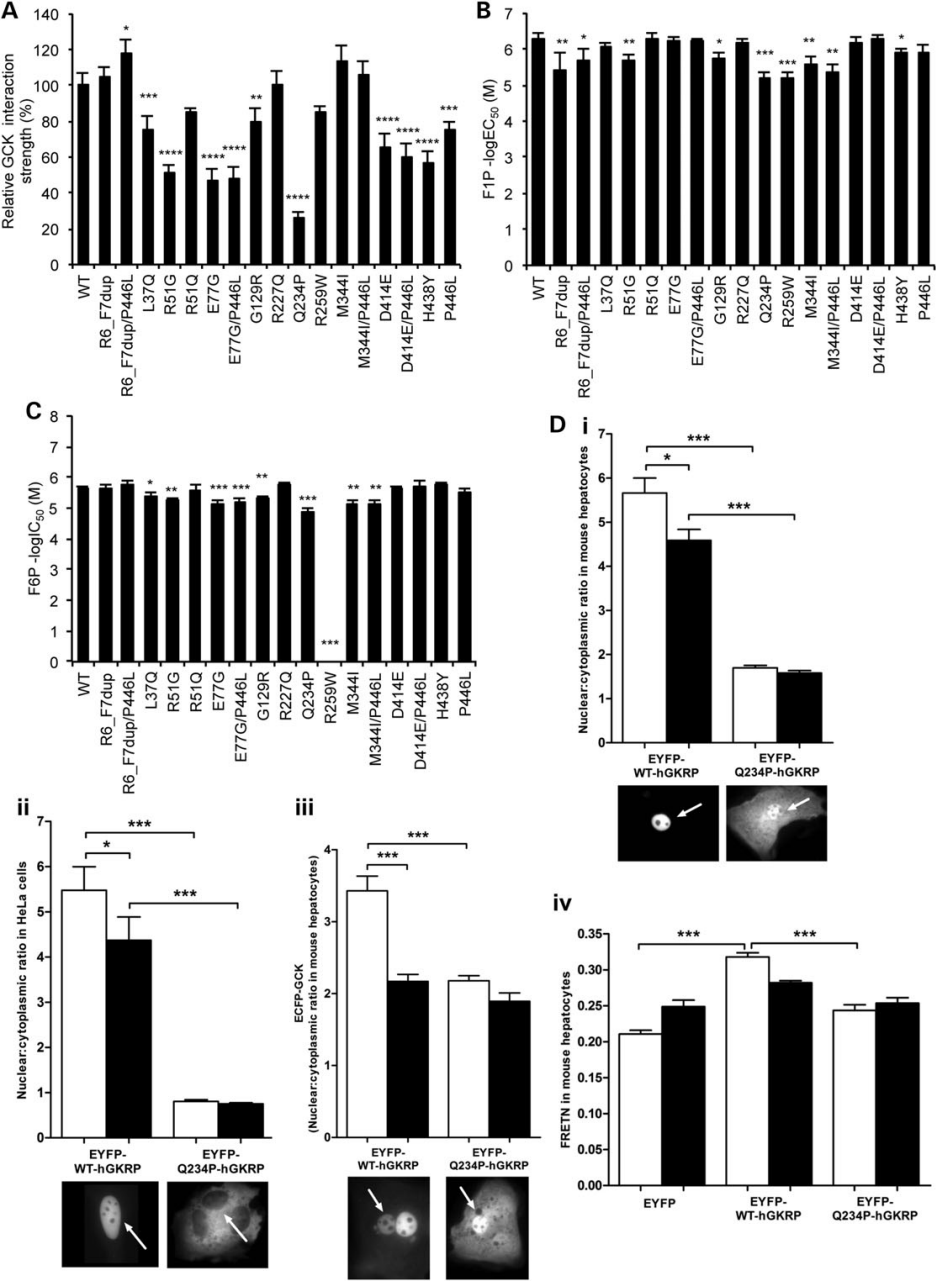
Functional properties of GKRP variants. Comparison of the affinity of recombinant WT or variant GKRP proteins for GCK via homogenous time-resolved fluorescence (**A**), and F1P (**B**) and F6P (**C**) via microscale thermophoresis. Interaction strength for each variant is depicted as a percentage of WT GKRP in (A) and as a negative log half-maximal effective concentration (B) or inhibitory concentration (C). An F6P dose–response curve could not be reliably fit for R259W because it did not appreciably respond to F6P (see Supplementary Material, Fig. S2B). Validation of high-throughput results using transient transfection-based cellular assays for the representative variant Q234P is shown in (**D**). This variant was tested for GKRP fluorescence localization in mouse hepatocytes (i) and HeLa cells (ii), effect on GCK fluorescence localization in mouse hepatocytes (iii) and GKRP–GCK interaction strength via quantitative fluorescence resonance energy transfer (FRETN) in mouse hepatocytes (iv). Results shown are mean ± SD of three to four replicates using two independent protein preparations per variant (A–C), and mean ± SEM in (D) (*n* = 3). White and black bars in (D), 5.5 mmol/l and 25 mmol/l glucose, respectively. The nucleus is indicated with an arrow. *P*-values *<0.05, **<0.01, ***<0.001 (ANOVA/Bonferroni correction).

**Figure 3.**
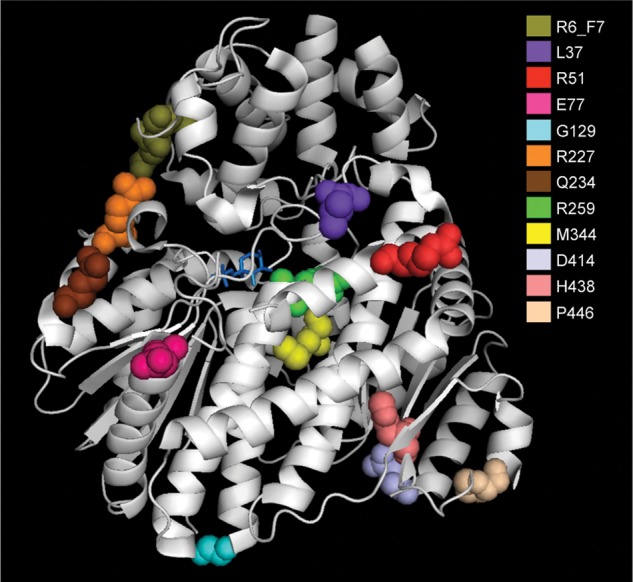
Ribbon model of the F1P-bound form of human GKRP. The structural model (Protein DataBank Entry 4BB9) indicates the location of each of the 10 missense variants, the insertion variant p.R6_F7dup and the common variant p.P446. F1P is indicated in stick form in blue.

The primary recognized function of GKRP is to interact with and inhibit the glucose sensor GCK in the hepatocyte nucleus. We expressed and purified recombinant human GCK and wild-type (WT) and variant human GKRP to explore the effects of rare *GCKR* variants on the GCK–GKRP interaction. In an effort to increase sensitivity and throughput relative to existing kinetic assays, and to enable robust comparison between variants, we simultaneously measured the interaction between GCK and each variant GKRP (including the common p.P446L variant) using a sensitive, quantitative and miniaturized homogenous time-resolved fluorescence (HTRF) assay (Fig. 2A). All five variants which showed reduced protein levels in Figure 1A also demonstrated significantly reduced interaction with GCK. The GCK–GKRP interaction is enhanced by binding of the glycolytic intermediate fructose-6-phosphate (F6P) to GKRP, and is reduced by binding of fructose-1-phosphate (F1P). The additional effect of both phosphate esters on the GCK–GKRP interaction in the HTRF assay was therefore measured (Supplementary Material, Figs S1 and S2A), as was the direct affinity of variant GKRPs for F1P and F6P in the absence of GCK using microscale thermophoresis (MST) (Fig. 2B and C, Supplementary Material, Fig. S2B). While this manuscript was in preparation, the crystal structure of human GKRP complexed with F1P was solved (11). Mapping of each variant within this structure supported our experimental findings, with those variants in closest proximity to the sugar-binding site (p.R259W, p.M344I) showing the most pronounced effects on F1P and F6P binding affinity (Fig. 3).

Significant defects were observed for most variants (10 of 12) for GCK interaction, F1P interaction and/or F6P interaction. Defective variants were further categorized as ‘Mild’ or ‘Severe’ based on the cumulative extent of dysfunction observed for each variant protein (Supplementary Material, Tables S1 and S2). These results were validated using an extensive repertoire of published kinetic and cell-based methodologies (Fig. 2D; Supplementary Material, Table S3 and Fig. S3) (3,9,10). For example, p.Q234P, p.M344I and p.H438Y all demonstrated a significant reduction in nuclear localization compared with WT-GKRP in both HeLa cells and primary mouse hepatocytes (Fig. 2D; Supplementary Material, Fig. S3). However, the degree of this cellular defect was most pronounced for p.Q234P, the only variant which demonstrated global defects in protein expression, GCK interaction, F1P affinity and F6P affinity (Figs 1A and 2A–C). Consistent with previous observations (3), the presence of the p.P446L variant in *cis* with rare variants augmented functional defects, most notably by decreasing nuclear localization and protein levels (Fig. 1B; Supplementary Material, Fig. S3).

To gauge the performance of mutation prediction algorithms, we compared our empirical findings with results from commonly used algorithms. SIFT human single-nucleotide polymorphisms (SNPs) (12) (6 of 11 correct predictions), PolyPhen-2 (13) (7 of 11) and Condel (14) (3 of 11) all performed relatively poorly on this data set (Table 1). The consequence of poor *in silico* prediction on gene-based association tests was apparent when we investigated the clinical phenotypes of *GCKR* variant carriers classified according to *in vitro* or *in silico* dysfunction (Tables 1 and 2). Individuals with *in vitro* deleterious *GCKR* variants were observed at higher frequency in hypertriglyceridemia cases compared with controls (4.1 versus 1.2%, *P* = 0.03, two-tailed Fisher's exact test; Table 2). When this analysis was repeated using *in silico* prediction as a variant classifier, only PolyPhen-2 predicted that deleterious *GCKR* variants were associated with hypertriglyceridemia. However, PolyPhen-2 results led to underestimation in the frequency of deleterious *GCKR* variants in cases (2.3%). Additionally, when considering only *GCKR* missense variants—those for which accurate *in silico* predictions are most necessary—the association between PolyPhen-2 predictions and hypertriglyceridemia was weakened (*P* = 0.14).

**Table 1.** Comparison of functional defects with bioinformatic predictions of human missense variant severity

Amino acid substitution	SIFT Human SNPs	PolyPhen-2	Condel	Functional defect(s) (Prot, GCK, F1P, F6P)	Overall functional assessment	Agreement
p.L37Q	Damaging	Probably damaging	Deleterious	XX-X	Damaging	All
p.R51G	Tolerated	Possibly damaging	Neutral	-XXX	Damaging	P
p.R51Q	Tolerated	Benign	Neutral	----	Benign	All
p.E77G	Damaging	Benign	Neutral	-X-X	Damaging	S
p.G129R	Damaging	Probably damaging	Neutral	XXXX	Damaging	S, P
p.R227Q	Tolerated	Probably damaging	Neutral	----	Benign	S, C
p.Q234P	Tolerated	Benign	Neutral	XXXX	Damaging	None
p.R259W	Damaging	Probably damaging	Neutral	--XX	Damaging	S, P
p.M344I	Tolerated	Benign	Neutral	--X-	Damaging	None
p.D414E	Tolerated	Possibly damaging	Neutral	-X--	Damaging	P
p.H438Y	Tolerated	Probably damaging	Neutral	XXX-	Damaging	P
p.A519T	Damaging	Probably damaging	Deleterious	N/A	N/A	N/A

*GCKR* (RefSeq NM_001486.3) variants were assessed using the SIFT Human SNPs, PolyPhen-2 version 2.2.2 and Condel webserver algorithms. Functional defect(s) indicate similarity to (−) or significant differences from (X) WT-GKRP in Figure 1A and Figure 2A–C. Functional assessment was assigned as ‘Benign’ for variants showing WT-like cellular and kinetic properties and ‘Damaging’ for variants with cellular and/or kinetic defects. Prot, protein expression assay; GCK, HTRF GKRP–GCK interaction assay; F1P, F1P thermophoresis assay; F6P, F6P thermophoresis assay; S, SIFT Human SNPs; P, PolyPhen-2; C, Condel. N/A, variant not assessed for functional properties because it travelled in *cis* with the upstream frameshift variant p.S183CfsX34.

**Table 2.** Phenotypic and functional classification of *GCKR* rare variant carriers

	HTG cases		Controls	
*GCKR* WT	419		327	
*GCKR* rare variant carriers	19		5	
Functional classes	Deleterious	WT-like	Deleterious	WT-like
*In vitro*, observed	18	1	4	1
SIFT, predicted^a^	8	10	1	4
PolyPhen-2, predicted^a^	10	8	1	4
Condel, predicted^a^	7	11	1	4

Clinical analysis was restricted to individuals of self-described European ancestry from the hypertriglyceridemia (HTG) sequencing cohort described previously (8). Individuals were first classified according to *GCKR* variant status (*GCKR* WT, individuals with no rare *GCKR* variants; *GCKR* rare variant carriers, individuals carrying one or more *GCKR* rare variant). *GCKR* rare variant carriers in cases and controls were further subdivided according to either observed *in vitro* dysfunction or predicted *in silico* dysfunction.

^a^Excludes variant R6_F7dup.

**Figure 4.**
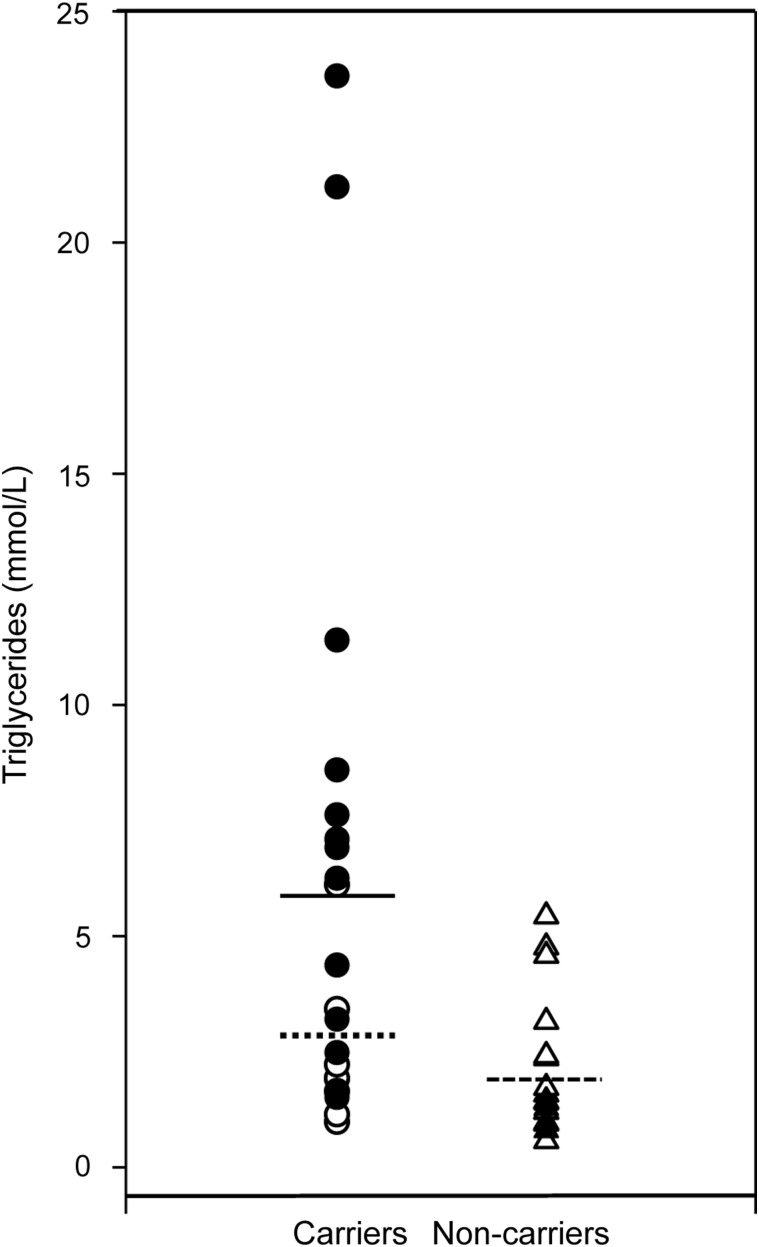
Phenotypic characteristics of *GCKR* rare variant carriers and non-carriers. Black circles, previously acquired carriers; white circles, newly acquired carriers; solid line, mean of previously acquired carriers; dotted line, mean of newly acquired carriers; black triangles, previously acquired non-carriers; white triangles, newly acquired non-carriers; dashed line, mean of non-carriers. *P*-values were calculated using unpaired *t*-tests assuming unequal variances between groups.

As our functional results supported the collective phenotypic relevance of rare *GCKR* variants that influence protein function, we aimed to address on an individual basis whether functional rare variants segregated with a hypertriglyceridemic phenotype by investigating their inheritance within families. Analysis of co-segregation of some rare variants with triglycerides in pedigrees could not be performed for various reasons, including the decision to decline participation and loss of individuals to follow-up. Ultimately, 16 of 24 original probands were available for follow-up, and an additional 25 family members were recruited across 12 of these probands. Rare variant and p.P446L status were determined by sequencing. In line with sample ascertainment, *GCKR* rare variant carriers had higher triglyceride and total cholesterol levels than non-carriers. However, newly ascertained variant carriers did not have significantly different triglyceride levels to newly ascertained non-carriers (1.8 ± 0.9 and 2.0 ± 1.4 mmol/l, respectively; mean ± SD; Fig. 4; Supplementary Material, Table S4). Although sufficient information was not available to draw decisive conclusions for every pedigree (e.g. p.M344I), *GCKR* variants generally did not co-segregate with raised triglyceride levels (Supplementary Material, Fig. S4). Individuals were also genotyped for the 10 common variants most strongly associated with plasma triglyceride levels from the Global Lipids Genetics Consortium to create both weighted and non-weighted genotype risk scores (15). Individuals with hypertriglyceridemia have been shown to have a significantly increased score (16). However, common variant burden scores performed no better than *GCKR* variant status in predicting triglyceride levels in this family-based data set (Supplementary Material, Fig. S4 and Table S4).

## DISCUSSION

Two major related challenges in the characterization of rare variants in complex disease are defining their functional effects and establishing their phenotypic consequences. *GCKR* serves as a useful model for the investigation of both of these issues. We utilized several reproducible and sensitive assays, both biochemical and cell biological, to investigate the effects of non-synonymous *GCKR* variants first identified in individuals with hypertriglyceridemia, including novel assays to measure variants simultaneously in a high-throughput manner (Fig. 2; Supplementary Material, Fig. S1), which provided a highly sensitive platform to compare the effects of different variants. Notably, functional results often did not agree with computational prediction algorithms; the use of *in silico* predictions alone would have resulted in a dramatically different interpretation of the role of *GCKR* variants in hypertriglyceridemia (Tables 1 and 2). Our results demonstrated a modest association between *GCKR* variants with *in vitro* dysfunction and hypertriglyceridemia (Table 2). However, currently, most large-scale sequencing or genotyping studies rely upon mutation prediction algorithms to assign functionality to non-synonymous variants. Consistent with emerging results from other comprehensive functional studies (2,3), three of the most commonly used prediction algorithms performed relatively poorly on this subset of *GCKR* variants (Table 1). Interestingly, the program with lowest accuracy, Condel, combines predictions across multiple algorithms, which some observers have suggested will increase accuracy (14). No algorithm performed appreciably better for the most deleterious variants, as some of the most functionally impaired variants across individual (e.g. p.M344I in phosphate ester assays) or all (e.g. p.Q234P) assays were not reliably predicted.

The comprehensive functional data available for *GCKR*, in combination with robust genetic data, also facilitated detailed phenotypic investigation. Associations with plasma triglyceride levels have been observed for the common p.P446L non-synonymous variant (7,17), and through collective analysis of functionally characterized rare variants in sample sets with both largely random (3) and highly selected ascertainment (Fig. 4 and Supplementary Material, Table S4). However, despite evidence that the variants tested are functionally and phenotypically relevant, rare variants showed little evidence of co-segregation with triglyceride levels within families (Supplementary Material, Fig. S4). Moreover, genotype risk scores for common variants influencing triglyceride levels did not clarify these discrepancies (Supplementary Material, Fig. S4 and Table S4). Therefore, in contrast to classical family-based studies in monogenic diseases, we found no evidence that functional *GCKR* rare variants have deterministic effects on plasma triglycerides in individuals and families, and their value as predictors of triglyceride levels in individuals in this data set is minimal.

The modest effect of *GCKR* variants on triglyceride levels in family members may also be relevant to the current controversy surrounding the potential effects of pharmacological GCK activation. GCK activators (GKAs) are currently in clinical trials as a potential treatment for Type 2 diabetes; however, these molecules may reduce plasma glucose concentrations at the expense of increased hepatic triglyceride biosynthesis (18–20). Although recent findings in rodent models and clinical trials suggest that GKA treatment may increase triglyceride levels, unfavorable lipid profiles have not been observed in individuals with activating *GCK* mutations (21–24). Our results suggest that indirect activation of hepatic GCK via loss-of-function *GCKR* mutations does not consistently lead to a hyperlipidemic phenotype, and are particularly relevant to the recent report of a selective small-molecule inhibitor of GKRP, which was shown to disrupt the GCK–GKRP interaction *in vivo* and reduced blood glucose levels in mouse models of obesity without apparent adverse effects on triglyceride levels (25). Continued exploration of the combination of genetic and clinical data with highly sensitive biochemical, cell-biological and animal model systems may provide further insight into the effects of pharmacological and genetic modulation of GKRP (25–27).

Collectively, our observations emphasize a complex inheritance pattern for plasma triglycerides, suggesting that other rare genetic and/or environmental factors may mitigate genetic load in particular individuals. Accordingly, while *GCKR* variation is associated with alterations in triglyceride levels, such variants are neither necessary nor sufficient to cause hypertriglyceridemia. Therefore, analysis of large numbers of carriers of individual rare variants may be required to ascertain the phenotypic effects of such variants, either through collection of extended pedigrees for private variants or through a combination of large-scale sequencing and/or segregation analyses for rare but non-unique variants. These results highlight the difficulty in translating findings arising from sequencing studies to phenotypic information on an individual level, even when molecular function can be determined for individual variants and additional variant carriers identified through family-based studies. As many rare variants discovered in future studies are likely to be novel, such challenges will only become more pronounced, particularly in light of the current reliance on *in silico* prediction algorithms and questions about their reliability. Studies of rare variants in other genes and complex traits should reveal the generalizability of this phenomenon.

## MATERIALS AND METHODS

### Ethical statement

Study subjects were unrelated hypertriglyceridemic probands determined to be heterozygous carriers of *GCKR* (RefSeq NM_001486.3) mutations by sequencing (8). Families were then extended for further assessments, including co-segregation of *GCKR* mutations with plasma triglyceride phenotype. The protocol was approved by the University of Western Ontario Health Sciences Research Ethics Board (protocol 07920E).

### Clinical and laboratory analyses

All study subjects had a complete medical history and physical examination was performed by the same clinician. Fasting plasma was drawn for the determination of concentrations of plasma lipids, lipoproteins and apolipoproteins, and for secondary causes of dyslipidemia. DNA was isolated from peripheral blood leucocytes, and the *GCKR* gene was sequenced using reagents and conditions as previously reported (8). For comparisons of clinical phenotypes, *P*-values were calculated using unpaired *t*-tests assuming unequal variances between groups. Unweighted polygenic risk score for hypertriglyceridemia was determined by tallying the number of triglyceride-raising alleles (0, 1 or 2) from genotypes of the top 10 triglyceride-associated SNPs from the Global Lipids Genetics Consortium (GLGC) (15), namely: *APOA5* rs964184, *GCKR* rs1260326, *LPL* rs12678919, *MLXIPL* rs7811265, *TRIB1* rs2954029, *APOB* rs1042034, *ANGPTL3* rs2131925, *APOE* rs439401, *CILP2* rs10401969 and *FADS1-2-3* rs174546. The weighted polygenic risk score was determined by multiplying the allele score (0, 1 or 2) at each of the top 10 loci by the respective beta-coefficient for triglyceride—raising from GLGC (15) at the associated locus, and then summing these. SAS version 9.3 (SAS Institute, Cary, NC, USA) was used for all statistical analyses.

### Cloning of *GCKR* and *GCK* fusion plasmids

#### Fluorescent fusion plasmids

Generation of human enhanced cyan fluorescent protein (ECFP)-tagged *GCK* and enhanced yellow fluorescent protein (EYFP)-tagged *GCKR* has been described previously (10,28).

#### Affinity tag fusion plasmids

Generation of GST-tagged *GCK* and FLAG-tagged *GCKR* bacterial expression vectors has been described previously (9,29). Myc-DDK-tagged *GCKR* in the pCMV6 mammalian expression vector was obtained from Origene (Cedarlane Laboratories).

### Mutagenesis

*GCKR* mutations were introduced via site-directed mutagenesis using the Stratagene QuikChange II, QuikChange II XL or QuikChange Lightning kit (Agilent Biotechnologies) according to the manufacturer's instructions. All plasmid sequences were verified by sequencing. All primers were obtained from Integrated DNA Technologies or Eurofins Genetic Services Ltd. Primer sequences are available upon request.

### Cell culture and transfection

For analysis of protein expression levels via western blotting, COS-7 cells were cultured in Dulbecco's Modified Eagle's Medium containing 50 units/ml penicillin and 50 µg/ml streptomycin, supplemented with 10% fetal bovine serum and maintained at 37°C/5% CO_2_. Cells were seeded at a density of 6 × 10^5^ per 60 mm dish the day before transfection. One microgram WT or variant *GCKR* plasmid DNA was transfected using Effectene Transfection Reagent and Enhancer (Qiagen) as per the manufacturer's instructions.

Mouse hepatocytes and HeLa cells were isolated, cultured and transfected with ECFP-GCK and EYFP-GKRP expression plasmids as described previously (28).

### Western blot analysis

Forty-eight hours post-transfection, cells were washed twice with PBS and lysed in sodium dodecyl sulfate (SDS) buffer (50 mm Tris–Cl pH 6.8, 2% SDS, 10% glycerol) that had been preheated at 95°C for 5 min. Samples were then incubated at 95°C for 5 min. Protein concentrations of whole cell lysates were determined using the DC Protein Assay kit (Bio-Rad). Lysates were supplemented with 50 mm Tris–Cl pH 6.8, 100 mm DTT, 2% SDS, 0.1% Bromophenol blue and 10% glycerol and heated to 95°C for 5 min before separation on a 4–12% gradient SDS–PAGE gel (Invitrogen). Samples were then transferred onto PVDF (Invitrogen) in a Bio-Rad transfer module. Blocking and hybridization were performed with anti-Myc monoclonal antibody (1:1000 dilution) or anti-DDK monoclonal antibody (1 : 2000 dilution, both from Origene Technologies) overnight. Signals were visualized using the SuperSignal West Pico Chemiluminescent Substrate kit (Pierce Biotechnology). Membranes were washed with Tris-buffered saline containing 0.1% Tween-20, stripped with stripping buffer (25 mm glycine, 1% SDS, pH 2.0) and hybridized to anti-tubulin antibody (1:25 000 dilution, Sigma-Aldrich), which was used as a loading control. For each variant and WT *GCKR* clone, the amount of Myc-DDK-tagged protein was measured and normalized to alpha-tubulin via densitometry. All normalized variant expression values were compared with WT GKRP as the reference standard using an unpaired (two-tailed) *t*-test.

### Fluorescence microscopy and image analysis

FRET experiments and calculation of GCK and GKRP nuclear: cytoplasmic ratios in primary mouse hepatocytes and HeLa cells were performed as described previously (28). Statistical analyses were performed by analysis of variance (ANOVA) followed by Bonferroni's test for multiple comparison.

### Functional characterization

#### Protein production and purification

Recombinant GST-tagged GCK and WT and variant FLAG-tagged GKRP proteins were prepared as described previously (9,29).

#### HTRF assays

HTRF technology was utilized for antibody-based FRET measurements of cell-free protein–protein interactions between GCK and WT or variant GKRP. Recombinant human GST-tagged GCK and WT or variant FLAG-tagged GKRP were mixed in an assay buffer (2 mm MgCl_2_, 25 mm KCl, 25 mm HEPES pH 7.1, 1 mm DTT, 0.025% BSA) identical to that used for kinetic assays (see below), with the exclusion of GCK substrates and coupling components and addition of 0.1% Tween-20. F1P or F6P was tested in an 11-point titration (half-log serial dilutions) with a maximum concentration of 3.16 mm or 1.39 mm, respectively. The reaction mixture was dispensed (3.5 µl/well) using an Aurora Discovery BioRAPTR Flying Reagent Dispenser (BioRAPTR; Beckman Coulter, Inc.) (30) into white 1536-well solid-bottom medium binding plates (Greiner Bio-One). Anti-FLAG XL 665 FRET acceptor- and anti-GST K FRET donor-conjugated antibodies (Cisbio Bioassays) were diluted to 4.8 ng/µl and 0.648 ng/µl, respectively, in a reconstitution buffer (1.6 m KF and 100 mm sodium phosphate, pH 7.0; both Sigma-Aldrich), and added to the plate using a BioRAPTR (0.5 µl/well). Negative control wells included all assay components except GKRP. Plates were lidded and incubated for 60 min at room temperature before being read on an Envision 2104 Multilabel Plate Reader (PerkinElmer) with an excitation at 320 nm and dual-wavelength detection at the FRET donor wavelength (615 nm) and FRET acceptor wavelength (665 nm). Statistical analyses were performed by ANOVA followed by Bonferroni's test for multiple comparison.

#### MST measurements

Binding kinetics of F1P and F6P to WT and variant FLAG-tagged GKRP were measured by MST in a NanoTemperMonolith NT.115 LabelFree instrument (NanoTemper Technologies). MST detects changes in size, charge and/or solvation induced by binding by measurement of intrinsic protein fluorescence (31). MST experiments were carried out in an HTRF assay buffer excluding BSA, with a final concentration of 250 nm WT or variant GKRP and F1P or F6P in 16-point two-fold serial dilution with maximum concentrations of 1 mm or 0.2 mm, respectively. Proteins were loaded into Standard Treated NT.LabelFree Capillaries (NanoTemper Technologies) and MST was measured using 15% LED power and 40% MST laser power.

#### Kinetic characterization

GKRP inhibition of GCK activity was determined spectrophotometrically using glucose-6-phosphate dehydrogenase-linked assays as described previously (9). F1P and F6P assays (0–500 µm) were also performed as described previously (9). F1P was purchased from Santa Cruz Biotechnology and F6P from Sigma-Aldrich. Statistical analyses were performed using Student's *t*-test.

### Variant classification

Variants were classified as ‘WT-like’, ‘Mild’ or ‘Severe’ based on their relative interaction strength with GCK via HTRF, and F1P and F6P via MST. The cut-offs for classification in each category are given in Supplementary Material, Table S2. A variant's overall classification took into account its cumulative performance in all three categories according to the following: ‘WT-like’, WT-like in all three categories; ‘Mild’, mild in at least one category and either WT-like or Mild in the other two; ‘Severe’, severe in at least one category.

### Graphical analyses and nonlinear regression

Nonlinear regression was utilized to generate dose–response curves for F1P and F6P using GraphPad Prism (GraphPad Software, San Diego, CA, USA). Thermophoresis was measured after the temperature jump using the ‘Thermophoresis, no jump’ option in the MST software (31). Modulator concentrations were converted to logarithmic values and the log(agonist) versus response (three parameters) or log(inhibitor) versus response (three parameters) options utilized to fit curves and determine the half-maximal effective concentration (EC_50_) or half-maximal inhibitory concentration (IC_50_).

### Structural modeling

Variants were mapped onto the crystal structure of human GKRP bound to F1P (Protein DataBank entry 4BB9) using PyMOL v.0.99.

## SUPPLEMENTARY MATERIAL

Supplementary Material is available at *HMG* online.

## FUNDING

This work was supported in Oxford by the Wellcome Trust (grant number 095101/Z/10/Z); in Canada by operating grants to RAH from the Heart and Stroke Foundation of Canada (grant number T6606) and the Canadian Institutes for Health Research (grant numbers MOP13430, MOP79533); in Rostock by the German Diabetes Association (DDG); and at NIH by the Molecular Libraries Common Fund Program of the National Institutes of Health and grant number Z01 HG000024/HG/NHGRI NIH HHS/United States to FSC. Funding to pay the Open Access publication charges for this article was provided by the Wellcome Trust.

## Supplementary Material

Supplementary Data

## References

[1] Palotie A., Widen E., Ripatti S. (2013). From genetic discovery to future personalized health research. Nat. Biotechnol..

[2] Bonnefond A., Clement N., Fawcett K., Yengo L., Vaillant E., Guillaume J.L., Dechaume A., Payne F., Roussel R., Czernichow S. (2012). Rare MTNR1B variants impairing melatonin receptor 1B function contribute to type 2 diabetes. Nat. Genet..

[3] Rees M.G., Ng D., Ruppert S., Turner C., Beer N.L., Swift A.J., Morken M.A., Below J.E., Blech I., Mullikin J.C. (2012). Correlation of rare coding variants in the gene encoding human glucokinase regulatory protein with phenotypic, cellular, and kinetic outcomes. J. Clin. Invest..

[4] Kiezun A., Garimella K., Do R., Stitziel N.O., Neale B.M., McLaren P.J., Gupta N., Sklar P., Sullivan P.F., Moran J.L. (2012). Exome sequencing and the genetic basis of complex traits. Nat. Genet..

[5] Flanagan S.E., Patch A.M., Ellard S. (2010). Using SIFT and PolyPhen to predict loss-of-function and gain-of-function mutations. Genet. Test. Mol. Biomarkers.

[6] Hunt S.C., Hasstedt S.J., Kuida H., Stults B.M., Hopkins P.N., Williams R.R. (1989). Genetic heritability and common environmental components of resting and stressed blood pressures, lipids, and body mass index in Utah pedigrees and twins. Am. J. Epidemiol..

[7] Saxena R., Voight B.F., Lyssenko V., Burtt N.P., de Bakker P.I., Chen H., Roix J.J., Kathiresan S., Hirschhorn J.N., Daly M.J. (2007). Genome-wide association analysis identifies loci for type 2 diabetes and triglyceride levels. Science.

[8] Johansen C.T., Wang J., Lanktree M.B., Cao H., McIntyre A.D., Ban M.R., Martins R.A., Kennedy B.A., Hassell R.G., Visser M.E. (2010). Excess of rare variants in genes identified by genome-wide association study of hypertriglyceridemia. Nat. Genet..

[9] Beer N.L., Tribble N.D., McCulloch L.J., Roos C., Johnson P.R., Orho-Melander M., Gloyn A.L. (2009). The P446L variant in GCKR associated with fasting plasma glucose and triglyceride levels exerts its effect through increased glucokinase activity in liver. Hum. Mol. Genet..

[10] Rees M.G., Wincovitch S., Schultz J., Waterstradt R., Beer N.L., Baltrusch S., Collins F.S., Gloyn A.L. (2012). Cellular characterisation of the GCKR P446L variant associated with type 2 diabetes risk. Diabetologia.

[11] Pautsch A., Stadler N., Lohle A., Rist W., Berg A., Glocker L., Nar H., Reinert D., Lenter M., Heckel A. (2013). Crystal structure of glucokinase regulatory protein. Biochemistry.

[12] Ng P.C., Henikoff S. (2001). Predicting deleterious amino acid substitutions. Genome Res..

[13] Adzhubei I.A., Schmidt S., Peshkin L., Ramensky V.E., Gerasimova A., Bork P., Kondrashov A.S., Sunyaev S.R. (2010). A method and server for predicting damaging missense mutations. Nat. Methods.

[14] Gonzalez-Perez A., Lopez-Bigas N. (2011). Improving the assessment of the outcome of nonsynonymous SNVs with a consensus deleteriousness score, Condel. Am. J. Hum. Genet..

[15] Teslovich T.M., Musunuru K., Smith A.V., Edmondson A.C., Stylianou I.M., Koseki M., Pirruccello J.P., Ripatti S., Chasman D.I., Willer C.J. (2010). Biological, clinical and population relevance of 95 loci for blood lipids. Nature.

[16] Johansen C.T., Wang J., Lanktree M.B., McIntyre A.D., Ban M.R., Martins R.A., Kennedy B.A., Hassell R.G., Visser M.E., Schwartz S.M. (2011). An increased burden of common and rare lipid-associated risk alleles contributes to the phenotypic spectrum of hypertriglyceridemia. Arterioscler. Thromb. Vasc. Biol..

[17] Orho-Melander M., Melander O., Guiducci C., Perez-Martinez P., Corella D., Roos C., Tewhey R., Rieder M.J., Hall J., Abecasis G. (2008). Common missense variant in the glucokinase regulatory protein gene is associated with increased plasma triglyceride and C-reactive protein but lower fasting glucose concentrations. Diabetes.

[18] Matschinsky F.M. (2009). Assessing the potential of glucokinase activators in diabetes therapy. Nat. Rev. Drug Discov..

[19] Nissim I., Horyn O., Daikhin Y., Wehrli S.L., Yudkoff M., Matschinsky F.M. (2012). Effects of a glucokinase activator on hepatic intermediary metabolism: study with (13)C-isotopomer-based metabolomics. Biochem. J..

[20] Rees M.G., Gloyn A.L. (2013). Small molecular glucokinase activators: has another new anti-diabetic therapeutic lost favour?. Br. J. Pharmacol..

[21] De Ceuninck F., Kargar C., Ilic C., Caliez A., Rolin J.O., Umbdenstock T., Vinson C., Combettes M., de Fanti B., Harley E. (2013). Small molecule glucokinase activators disturb lipid homeostasis and induce fatty liver in rodents: a warning for therapeutic applications in humans. Br. J. Pharmacol..

[22] Meininger G.E., Scott R., Alba M., Shentu Y., Luo E., Amin H., Davies M.J., Kaufman K.D., Goldstein B.J. (2011). Effects of MK-0941, a novel glucokinase activator, on glycemic control in insulin-treated patients with type 2 diabetes. Diabetes Care.

[23] Christesen H.B., Jacobsen B.B., Odili S., Buettger C., Cuesta-Munoz A., Hansen T., Brusgaard K., Massa O., Magnuson M.A., Shiota C. (2002). The second activating glucokinase mutation (A456 V): implications for glucose homeostasis and diabetes therapy. Diabetes.

[24] Gloyn A.L., Noordam K., Willemsen M.A., Ellard S., Lam W.W., Campbell I.W., Midgley P., Shiota C., Buettger C., Magnuson M.A. (2003). Insights into the biochemical and genetic basis of glucokinase activation from naturally occurring hypoglycemia mutations. Diabetes.

[25] Lloyd D.J., St Jean D.J., Kurzeja R.J., Wahl R.C., Michelsen K., Cupples R., Chen M., Wu J., Sivits G., Helmering J. (2013). Antidiabetic effects of glucokinase regulatory protein small-molecule disruptors. Nature.

[26] Kaminski M.T., Schultz J., Waterstradt R., Tiedge M., Lenzen S., Baltrusch S. (2013). Glucose-induced dissociation of glucokinase from its regulatory protein in the nucleus of hepatocytes prior to nuclear export. Biochim. Biophys. Acta.

[27] Rees M.G., Davis M.I., Shen M., Titus S., Raimondo A., Barrett A., Gloyn A.L., Collins F.S., Simeonov A. (2014). A Panel of Diverse Assays to Interrogate the Interaction between Glucokinase and Glucokinase Regulatory Protein, Two Vital Proteins in Human Disease. PLoS One.

[28] Baltrusch S., Francini F., Lenzen S., Tiedge M. (2005). Interaction of glucokinase with the liver regulatory protein is conferred by leucine–asparagine motifs of the enzyme. Diabetes.

[29] Liang Y., Kesavan P., Wang L.Q., Niswender K., Tanizawa Y., Permutt M.A., Magnuson M.A., Matschinsky F.M. (1995). Variable effects of maturity-onset-diabetes-of-youth (MODY)-associated glucokinase mutations on substrate interactions and stability of the enzyme. Biochem. J..

[30] Niles W.D., Coassin P.J. (2005). Piezo- and solenoid valve-based liquid dispensing for miniaturized assays. Assay Drug Dev. Technol..

[31] Seidel S.A., Wienken C.J., Geissler S., Jerabek-Willemsen M., Duhr S., Reiter A., Trauner D., Braun D., Baaske P. (2012). Label-free microscale thermophoresis discriminates sites and affinity of protein-ligand binding. Angew. Chem. Int. Ed. Engl..

