# Supplementary material for: Inheritance of rare functional GCKR variants and their contribution to triglyceride levels in families
Source: Hum Mol Genet. 2014 May 30;23(20):5570–8. doi: 10.1093/hmg/ddu269 (PMC4168830; doi:10.1093/hmg/ddu269)
Supplement: Supplementary Data [file supp_23_20_5570__index.html]

Inheritance of rare functional GCKR variants and their contribution to triglyceride levels in families — Inheritance of rare functional GCKR variants and their contribution to triglyceride levels in families — Supplementary Data 

# Inheritance of rare functional *GCKR* variants and their contribution to triglyceride levels in families

## Supplementary Data

Supplementary Data

**Files in this Data Supplement:**

- Supplementary Data - Pdf file
