# Supplementary material for: Inheritance of rare functional GCKR variants and their contribution to triglyceride levels in families
Source: Hum Mol Genet. 2014 May 30;23(20):5570–8. doi: 10.1093/hmg/ddu269 (PMC4168830; doi:10.1093/hmg/ddu269)
Supplement: Supplementary Data [file supp_ddu269_ddu269supp.pdf]

Figure S1. Affinity of recombinant GKRP proteins for F1P and F6P via HTRF.

Comparison of the affinity of recombinant GKRP proteins for fructose 1-phosphate (F1P) (A) and fructose 6-phosphate (F6P) (B) via homogenous time-resolved fluorescence. Interaction strength for each GKRP protein is depicted as a negative log half-maximal effective concentration (A) or inhibitory concentration (B). An F6P dose-response curve could not be reliably fit for R259W because it did not appreciably respond to F6P (see Figure S2A). Results shown are mean ± SD (n=4). P values * < 0.05, ** <0.01, *** <0.001, **** < 0.0001.

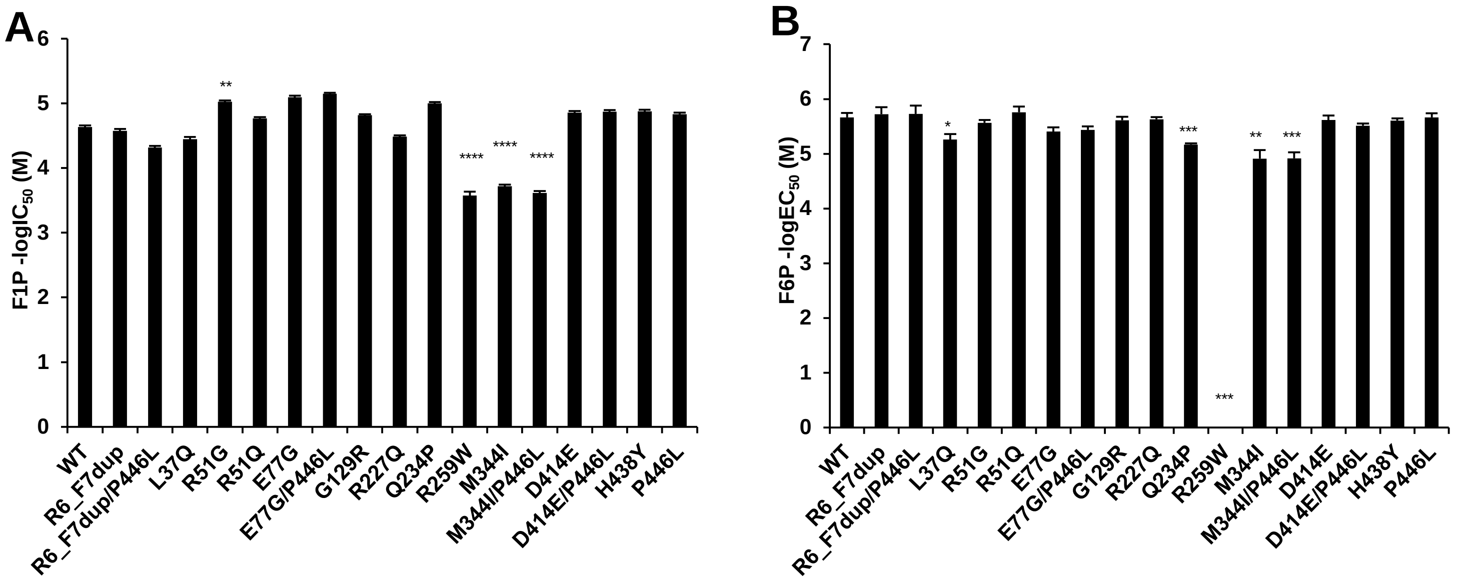
A
B
F1P -logIC50 (M)
F6P -logEC50 (M)
WT
R6_F7dup
R6_F7dup/P446L
L37Q
R51G
R51Q
E77G
E77G/P446L
G129R
R227Q
Q234P
R259W
M344I
M344I/P446L
D414E
D414E/P446L
H438Y
P446L

Figure S2. Fructose 6-phosphate (F6P) dose-response curves for WT- and R259W-GKRP.

Dose response curves were measured via homogenous time-resolved fluorescence (A) and microscale thermophoresis (B). Results shown are mean ± SD (n=4).

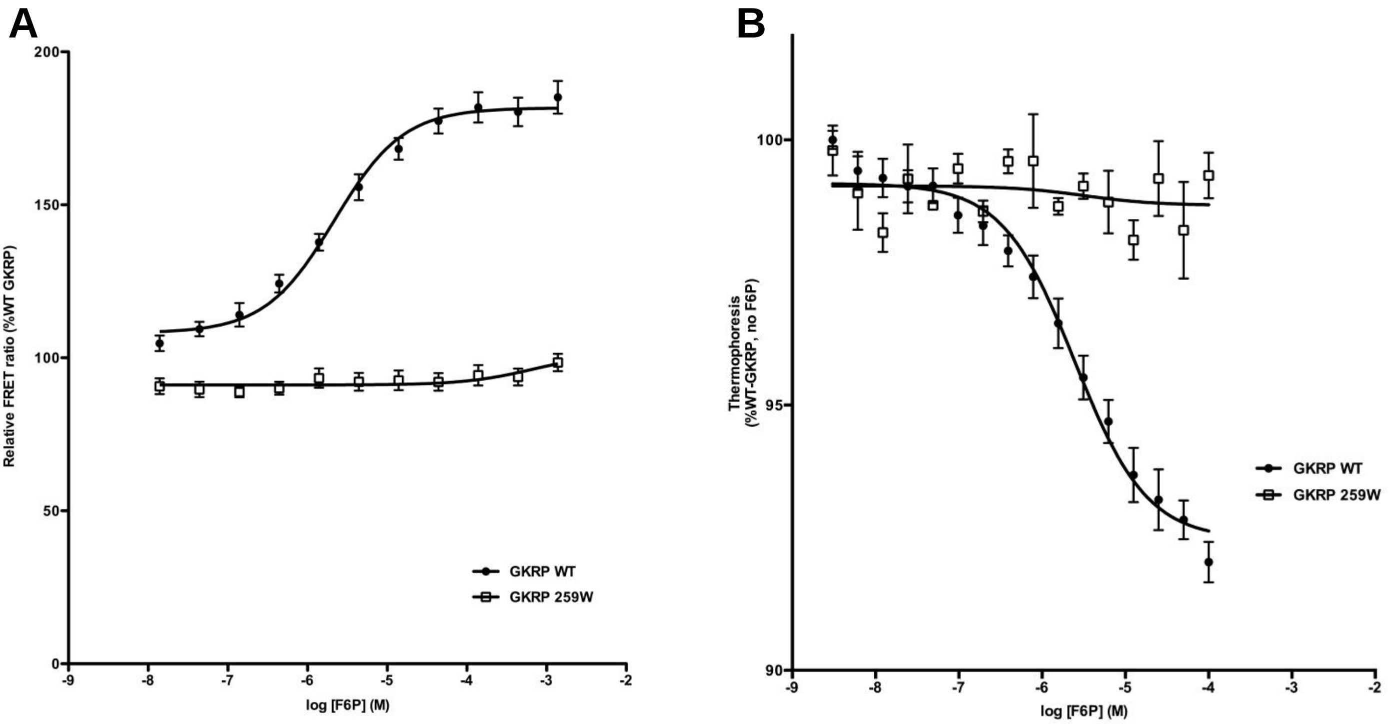
A
200
150
100
50
0
Relative FRET ratio (%WT GKRP)
-9
-8
-7
-6
-5
-4
-3
-2
log [F6P] (M)
GKRP WT
GKRP 259W
B
100
95
90
Thermophoresis
(%WT-GKRP, no F6P)
log [F6P] (M)

Figure S3. Validation of high-throughput results using additional cellular assays.

Variants were tested for GKRP fluorescence localization in mouse hepatocytes (A) and HeLa cells (B), effect on WT GCK fluorescence localization in mouse hepatocytes (C), and GKRP-GCK interaction strength via quantitative fluorescence resonance energy transfer (FRETN) in mouse hepatocytes (D). White bars, 5.5mmol/L glucose; black bars, 25mmol/L glucose. The nucleus is indicated with an arrow. Results shown are mean ±SEM (n=3). P values * <0.05, ** <0.01, *** <0.001.

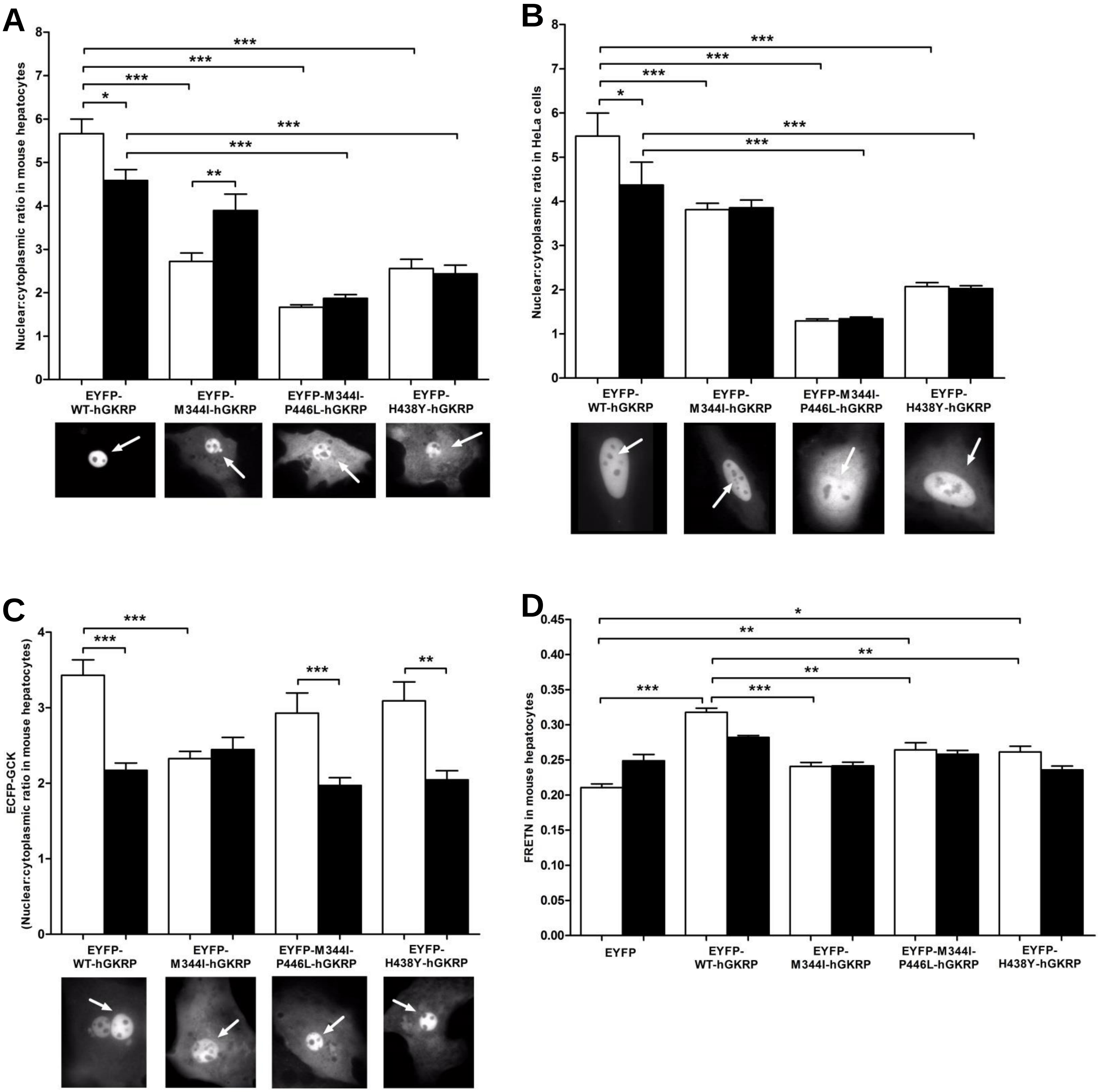
A
Nuclear:cytoplasmic ratio in mouse hepatocytes
EYFP-
WT-hGKRP
EYFP-
M344I-hGKRP
EYFP-M344I-
P446L-hGKRP
EYFP-
H438Y-hGKRP
B
Nuclear:cytoplasmic ratio in HeLa cells
C
ECFP-GCK
(Nuclear:cytoplasmic ratio in mouse hepatocytes)
D
FRETN in mouse hepatocytes
EYFP

Figure S4. Pedigrees for *GCKR* variant carriers.

Squares and circles denote males and females, respectively. Half-filled symbols represent heterozygous *GCKR* variant carriers, and unfilled symbols non-carriers. The proband is indicated with an arrow. BMI, body mass index; TC, total cholesterol; TG, triglycerides; LDL-C, low-density lipoprotein cholesterol; HDL-C, high-density lipoprotein cholesterol; FPG, fasting plasma glucose; GLGC, Global Lipids Genetics Consortium. N/A, information not available.

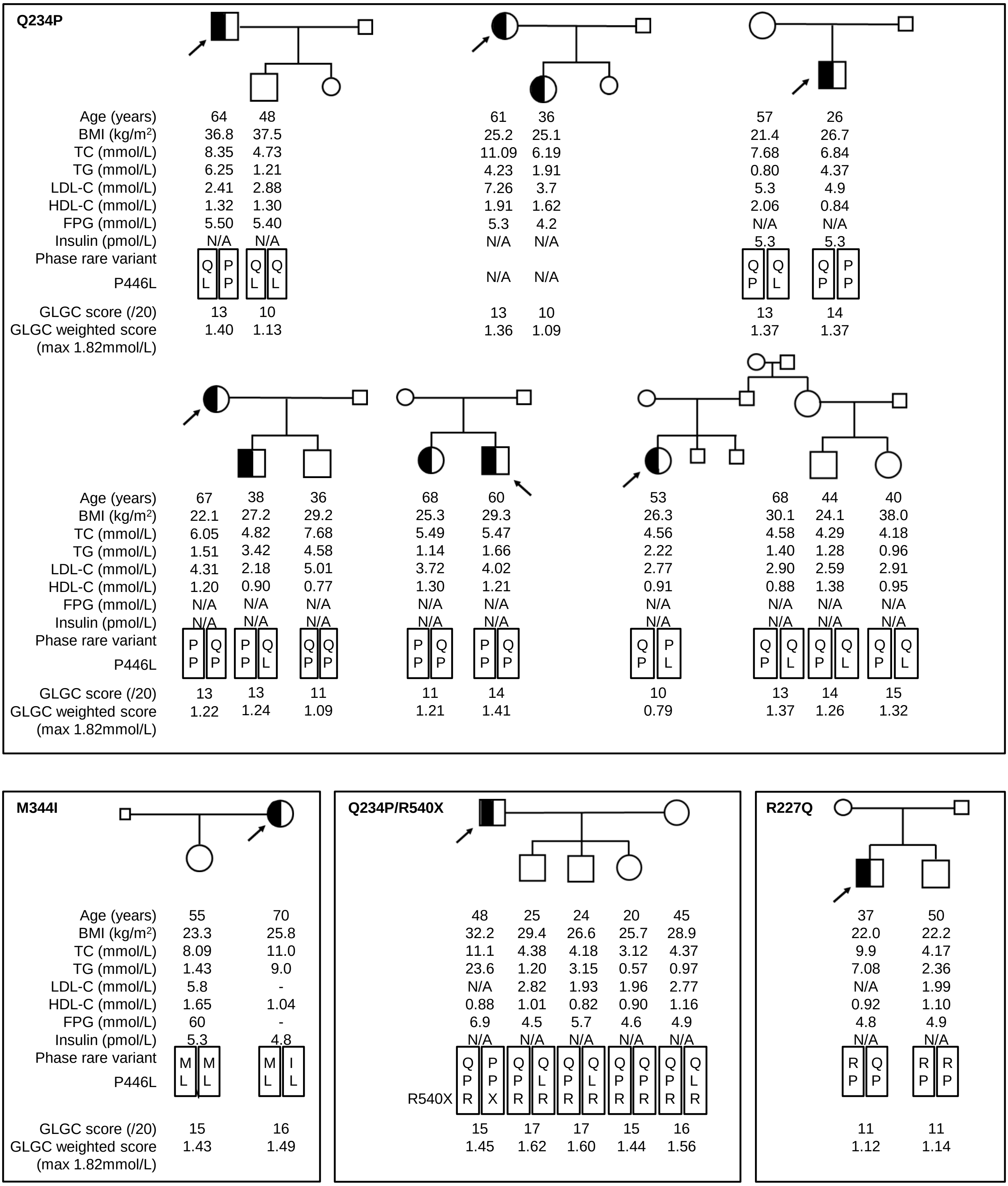

Q234P

Age (years)	64	48	61	36	57	26
BMI (kg/m^2)	36.8	37.5	25.2	25.1	21.4	26.7
TC (mmol/L)	8.35	4.73	11.09	6.19	7.68	6.84
TG (mmol/L)	6.25	1.21	4.23	1.91	0.80	4.37
LDL-C (mmol/L)	2.41	2.88	7.26	3.7	5.3	4.9
HDL-C (mmol/L)	1.32	1.30	1.91	1.62	2.06	0.84
FPG (mmol/L)	5.50	5.40	5.3	4.2	N/A	N/A
Insulin (pmol/L)	N/A	N/A	N/A	N/A	5.3	5.3
Phase rare variant	Q P	Q Q	N/A	N/A	Q Q	Q P
P446L	L P	L L			P L	P P
GLGC score (/20)	13	10	13	10	13	14
GLGC weighted score (max 1.82mmol/L)	1.40	1.13	1.36	1.09	1.37	1.37

Age (years)	67	38	36	68	60	53	68	44	40
BMI (kg/m^2)	22.1	27.2	29.2	25.3	29.3	26.3	30.1	24.1	38.0
TC (mmol/L)	6.05	4.82	7.68	5.49	5.47	4.56	4.58	4.29	4.18
TG (mmol/L)	1.51	3.42	4.58	1.14	1.66	2.22	1.40	1.28	0.96
LDL-C (mmol/L)	4.31	2.18	5.01	3.72	4.02	2.77	2.90	2.59	2.91
HDL-C (mmol/L)	1.20	0.90	0.77	1.30	1.21	0.91	0.88	1.38	0.95
FPG (mmol/L)	N/A	N/A	N/A	N/A	N/A	N/A	N/A	N/A	N/A
Insulin (pmol/L)	N/A	N/A	N/A	N/A	N/A	N/A	N/A	N/A	N/A
Phase rare variant	P Q	P Q	Q Q	P Q	P Q	Q P	Q Q	Q Q	Q Q
P446L	P P	P L	P P	P P	P P	P L	P L	P L	P L
GLGC score (/20)	13	13	11	11	14	10	13	14	15
GLGC weighted score (max 1.82mmol/L)	1.22	1.24	1.09	1.21	1.41	0.79	1.37	1.26	1.32

M344I

Age (years)	55	70
BMI (kg/m^2)	23.3	25.8
TC (mmol/L)	8.09	11.0
TG (mmol/L)	1.43	9.0
LDL-C (mmol/L)	5.8	-
HDL-C (mmol/L)	1.65	1.04
FPG (mmol/L)	60	-
Insulin (pmol/L)	5.3	4.8
Phase rare variant	M M	M I
P446L	L L	L L
GLGC score (/20)	15	16
GLGC weighted score (max 1.82mmol/L)	1.43	1.49

Q234P/R540X

Age (years)	48	25	24	20	45
BMI (kg/m^2)	32.2	29.4	26.6	25.7	28.9
TC (mmol/L)	11.1	4.38	4.18	3.12	4.37
TG (mmol/L)	23.6	1.20	3.15	0.57	0.97
LDL-C (mmol/L)	N/A	2.82	1.93	1.96	2.77
HDL-C (mmol/L)	0.88	1.01	0.82	0.90	1.16
FPG (mmol/L)	6.9	4.5	5.7	4.6	4.9
Insulin (pmol/L)	N/A	N/A	N/A	N/A	N/A
Phase rare variant	Q P	Q Q	Q Q	Q Q	Q Q
P446L	P P	P L	P L	P P	P L
R540X	R X	R R	R R	R R	R R
GLGC score (/20)	15	17	17	15	16
GLGC weighted score (max 1.82mmol/L)	1.45	1.62	1.60	1.44	1.56

R227Q

Age (years)	37	50
BMI (kg/m^2)	22.0	22.2
TC (mmol/L)	9.9	4.17
TG (mmol/L)	7.08	2.36
LDL-C (mmol/L)	N/A	1.99
HDL-C (mmol/L)	0.92	1.10
FPG (mmol/L)	4.8	4.9
Insulin (pmol/L)	N/A	N/A
Phase rare variant	R Q	R R
P446L	P P	P P
GLGC score (/20)	11	11
GLGC weighted score (max 1.82mmol/L)	1.12	1.14

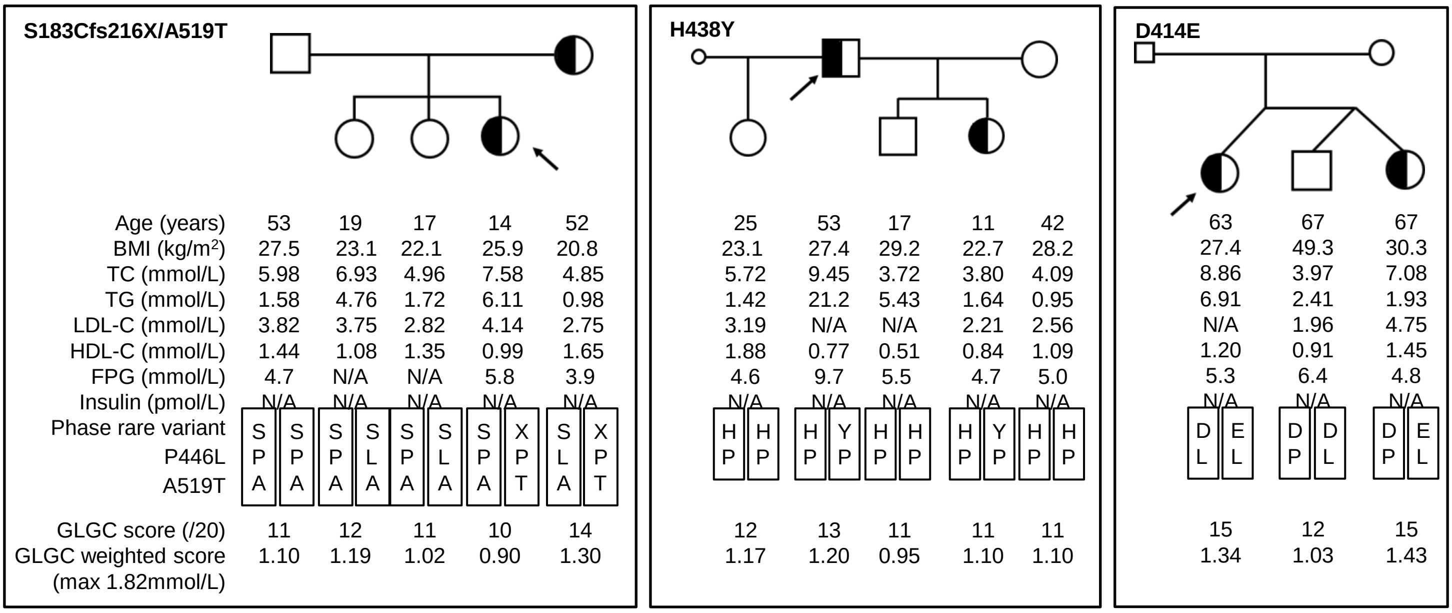
S183Cfs216X/A519T
H438Y
D414E
Age (years) 53 19 17 14 52 25 53 17 11 42 63 67 67
BMI (kg/m2) 27.5 23.1 22.1 25.9 20.8 23.1 27.4 29.2 22.7 28.2 27.4 49.3 30.3
TC (mmol/L) 5.98 6.93 4.96 7.58 4.85 5.72 9.45 3.72 3.80 4.09 8.86 3.97 7.08
TG (mmol/L) 1.58 4.76 1.72 6.11 0.98 1.42 21.2 5.43 1.64 0.95 6.91 2.41 1.93
LDL-C (mmol/L) 3.82 3.75 2.82 4.14 2.75 3.19 N/A N/A 2.21 2.56 N/A 1.96 4.75
HDL-C (mmol/L) 1.44 1.08 1.35 0.99 1.65 1.88 0.77 0.51 0.84 1.09 1.20 0.91 1.45
FPG (mmol/L) 4.7 N/A N/A 5.8 3.9 4.6 9.7 5.5 4.7 5.0 5.3 6.4 4.8
Insulin (pmol/L) N/A N/A N/A N/A N/A N/A N/A N/A N/A N/A N/A N/A N/A
Phase rare variant
P446L
A519T
SPA SPA | SPA SLA | SPA SLA | SPA XPT | SLA XPT
HP HP | HP YP | HP HP | HP YP | HP HP
DL EL | DP DL | DP EL
GLGC score (/20) 11 12 11 10 14 12 13 11 11 11 15 12 15
GLGC weighted score 1.10 1.19 1.02 0.90 1.30 1.17 1.20 0.95 1.10 1.10 1.34 1.03 1.43
(max 1.82mmol/L)

Table S1. Classification of *GCKR* variants according to functional effects.

Protein	F1P interaction	F6P interaction	GCK interaction	OVERALL CLASSIFICATION
p.R6_F7dup	Mild	WT-like	WT-like	MILD
p.R6_F7dup/P446L	Mild	WT-like	Mild	MILD
p.L37Q	WT-like	Mild	Mild	MILD
p.R51G	Mild	Mild	Severe	SEVERE
p.R51Q	WT-like	WT-like	WT-like	WT-LIKE
p.E77G	WT-like	Severe	Severe	SEVERE
p.E77G/P446L	WT-like	Severe	Severe	SEVERE
p.G129R	Mild	Mild	Mild	MILD
p.R227Q	WT-like	WT-like	WT-like	WT-LIKE
p.Q234P	Severe	Severe	Severe	SEVERE
p.R259W	Severe	Severe	WT-like	SEVERE
p.M344I	Mild	Severe	WT-like	SEVERE
p.M344I/P446L	Severe	Severe	WT-like	SEVERE
p.D414E	WT-like	WT-like	Severe	SEVERE
p.D414E/P446L	WT-like	WT-like	Severe	SEVERE
p.H438Y	Mild	WT-like	Severe	SEVERE
p.P446L	WT-like	WT-like	Mild	MILD

WT, wild-type; F1P, fructose 1-phosphate; F6P, fructose 6-phosphate.

Table S2. Cut-offs for variant classification according to functional effects.

	GCK interaction	F1P interaction	F6P interaction
WT-like	activity 85-110% of WT and $p \geq 0.05$	$p \geq 0.05$	$p \geq 0.05$
Mild	activity 70-85% or >110% of WT and $p<0.05$	>2-fold increase in EC_{50} and $p<0.05$	>1.5-fold increase in IC_{50} and $p<0.05$
Severe	activity <70% of WT and $p<0.0001$	>7-fold increase in EC_{50} and $p<0.001$	>2.5-fold increase in IC_{50} and $p<0.001$

Table S3. Validation of high-throughput results using additional kinetic assays.

Protein	1 Unit (µg)	P value	Relative amplitude of F1P response	P value	Relative amplitude of F6P response	P value
WT	5.12		1.00		1.00	
p.R6_F7dup	6.99	0.015	0.83 ± 0.05	0.04	1.12 ± 0.03	0.17
p.R6_F7dup/P446L	7.99	0.012	0.86 ± 0.03	0.05	1.12 ± 0.03	0.16
p.L37Q	5.01	0.128	1.08 ± 0.13	0.62	1.26 ± 0.10	0.12
p.R51G	7.23	0.005	1.02 ± 0.10	0.88	1.15 ± 0.04	0.06
p.R51Q	7.16	0.006	1.01 ± 0.11	0.94	1.05 ± 0.03	0.24
p.E77G	8.57	0.005	1.04 ± 0.08	0.69	1.47 ± 0.11	0.05
p.E77G/P446L	8.43	0.005	1.00 ± 0.01	0.68	1.29 ± 0.08	0.17
p.G129R	7.76	0.008	0.71 ± 0.07	0.004	1.08 ± 0.15	0.84
p.R227Q	5.21	0.733	0.94 ± 0.03	0.17	1.07 ± 0.05	0.26
p.Q234P	7.12	0.006	0.86 ± 0.03	<0.001	0.87 ± 0.07	0.05
p.R259W	8.11	0.002	0.79 ± 0.04	<0.001	0.50 ± 0.07	<0.001
p.M344I	5.21	0.417	0.33 ± 0.04	<0.001	0.94 ± 0.09	0.39
p.M344I/P446L	5.23	0.932	0.75 ± 0.04	0.03	0.79 ± 0.02	0.004
p.D414E	5.43	0.211	1.06 ± 0.01	0.10	0.83 ± 0.06	0.07
p.D414E/P446L	7.15	0.006	0.98 ± 0.01	0.29	0.99 ± 0.02	0.65
p.H438Y	6.26	0.005	0.92 ± 0.04	0.05	1.05 ± 0.11	0.82
p.P446L	8.21	0.005	0.93 ± 0.01	0.07	0.96 ± 0.03	0.81

1 Unit of GKRP is the amount required to achieve 50% inhibition of 10mU/mL WT-GCK (see Materials and Methods).

Table S4. Clinical and genetic attributes of *GCKR* rare variant carriers and non-carriers from family-based follow-up study.

	Carriers	Non-carriers	P value	Newly acquired carriers only
n	22	19		6
Female (%)	54.5	52.6	NS (0.91)	83.3
T2D (%)	27.3	10.5	NS (0.18)	0
Age (years)	50.1±17.6	39.6±16.8	NS (0.071)	45.3±21.4
BMI (kg/m^2)	29.5±10.5	28.8±6.9	NS (0.99)	25.3±3.3
TC (mmol/L)	7.0±2.1	5.1±1.5	0.0031	5.4±1.2
HDL-C (mmol/L)	1.1±0.4	1.2±0.5	NS (0.49)	1.3±0.4
LDL-C (mmol/L)	3.4±1.0	3.0±1.3	NS (0.38)	3.2±1.0
TG (mmol/L)	6.0±6.0	2.0±1.4	0.0019	1.8±0.9
Apo AI (g/L)	1.3±0.3	1.3±0.3	NS (0.68)	1.5±0.3
Apo B (g/L)	1.1±0.3	0.9±0.2	NS (0.15)	1.0±0.2
ALT (units/L)	45.5±47.5	30.5±12.4	NS (0.78)	15.8±1.3
AST (units/L)	35.0±22.3	27.3±9.2	NS (0.50)	20.8±2.4
HbA1c (mol/mol)	0.059±0.009	0.058±0.009	NS (0.80)	0.056±0.004
Lp(a) (mg/dL)	6.7±3.2	7.2±4.4	NS (0.69)	5.0±0.0
Fasting glucose (mmol/L)	5.6±1.4	5.1±0.5	NS (0.51)	4.4±0.4
C-reactive protein (mg/L)	4.2±4.4	3.3±2.0	NS (1.00)	3.1±1.7
Unweighted GLGC risk score (/20)	12.8±1.8	13.0±2.2	NS (0.86)	12.3±2.0
Weighted GLGC risk score (/1.8)	1.2±0.2	1.2±0.2	NS (0.90)	1.2±0.1
p.P446L frequency (%)	27.2	36.8	NS (0.52)	33.3

T2D, type 2 diabetes; BMI, body mass index; TC, total cholesterol; HDL-C, high-density lipoprotein cholesterol; LDL-C, low-density lipoprotein cholesterol; TG, triglycerides; Apo A1, apolipoprotein A1; Apo B, apolipoprotein B; ALT, alanine aminotransferase; AST, aspartate aminotransferase; HbA1c, haemoglobin A1C; Lp(a), lipoprotein(a). Values are mean ± SD.
